# The molecular underpinnings of fertility: Genetic approaches in *Caenorhabditis elegans*

**DOI:** 10.1002/ggn2.10034

**Published:** 2020-11-09

**Authors:** Xue Mei, Andrew W. Singson

**Affiliations:** ^1^ Department of Genetics Waksman Institute, Rutgers, The State University of New Jersey Piscataway New Jersey USA

**Keywords:** C. elegans, egg, fertility, fertilization, forward genetic screens, mutants, sperm, reproduction

## Abstract

The study of mutations that impact fertility has a catch‐22. Fertility mutants are often lost since they cannot simply be propagated and maintained. This has hindered progress in understanding the genetics of fertility. In mice, several molecules are found to be required for the interactions between the sperm and egg, with JUNO and IZUMO1 being the only known receptor pair on the egg and sperm surface, respectively. In *Caenorhabditis elegans*, a total of 12 proteins on the sperm or oocyte have been identified to mediate gamete interactions. Majority of these genes were identified through mutants isolated from genetic screens. In this review, we summarize the several key screening strategies that led to the identification of fertility mutants in *C*. *elegans* and provide a perspective about future research using genetic approaches. Recently, advancements in new technologies such as high‐throughput sequencing and Crispr‐based genome editing tools have accelerated the molecular, cell biological, and mechanistic analysis of fertility genes. We review how these valuable tools advance our understanding of the molecular underpinnings of fertilization. We draw parallels of the molecular mechanisms of fertilization between worms and mammals and argue that our work in *C*. *elegans* complements fertility research in humans and other species.

## Graphical abstract

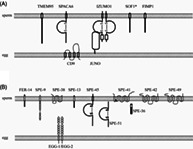

## 1 INTRODUCTION

**FIGURE 1:**
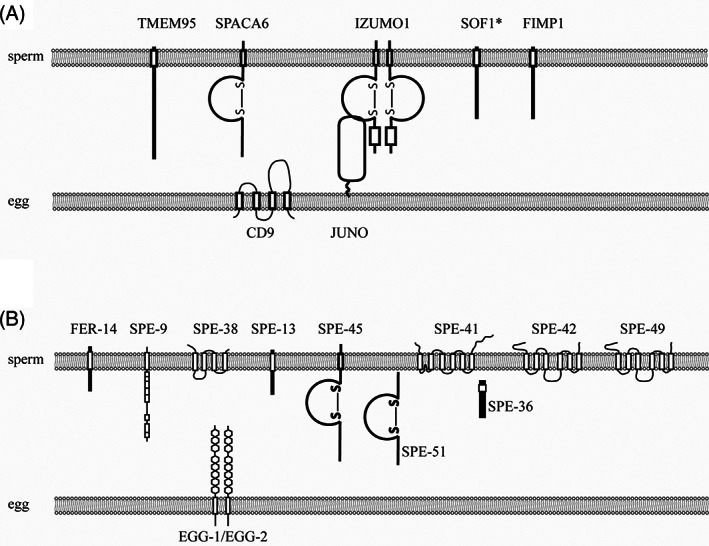
Currently known components of the mammalian and*C*.*elegans*fertilization synapse. A, Mammalian fertilization synapse. *, SOF1 is predicted to be a transmembrane protein. However, a few topology prediction programs predict it as a secreted protein (Personal communications with Dr. Ikawa and Dr. Noda and our own analyses). B,*C*.*elegans*fertilization synapse

A fundamental process during sexual reproduction, fertilization involves species‐specific recognition, adhesion and fusion between the gametes. These processes are thought to be mediated by molecular interactions between the gametes.^1^, ^2^ In mammals, the egg coat (also called zona pellucida) contains ZP proteins that are necessary and sufficient to support recognition.^1^, ^3^ Following recognition and penetration of the egg coat, several molecules are required for sperm‐egg binding and thus fusion, including the egg surface tetraspanin CD9 and the most recently identified sperm proteins FIMP, SOF1 and TMEM95 (Table 1 and Figure 1A). Among these molecules, the only known receptor‐binding pair is the Immunoglobulin (Ig) superfamily member IZUMO1 in the sperm and the Glycosylphosphatidylinositol (GPI)‐anchored JUNO in the egg.^4^, ^10^ In zebrafish, a GPI‐anchored protein called Bouncer is identified as an egg surface receptor that is necessary for species‐specific gamete interactions.^13^ However, the binding partner for Bouncer remains unknown. In *Caenorhabditis elegans*, the first molecule required for fertilization was discovered as SPE‐9, a transmembrane protein with EGF repeats, on the sperm surface. Mutants of *spe‐9* were reported in a forward genetic screen in 1988 and cloning of the gene was reported in 1998.^14^, ^17^ To date, a total of 12 *C*. *elegans* proteins have been found on the sperm and oocyte to mediate sperm‐oocyte interactions including an IZUMO1‐like molecule SPE‐45 (Table 1 and Figure 1B). Yet, among these molecules, no receptor pairs have been identified. What additional molecules are at play and how they interact with one another is unknown.

**TABLE 1:** Fertilization molecules in vertebrates and worms

Gene	Species/gamete	Protein domains/features	Reference
*Izumo1*	Mouse/sperm	Single‐pass TM protein with Ig‐like domain	4
*Spaca6*	Mouse/sperm	Single‐pass TM protein with Ig‐like domain	5, 6, and 7
*Tmem95*	Mouse/sperm	Single‐pass TM protein with secondary structures similar to the “IZUMO1” domain	5 and 6
*Fimp1*	Mouse/sperm	Single‐pass TM protein^a^ ^,^ ^b^	8
*Sof1*	Mouse/sperm	Protein with conserved “LLLL and CFNLAS” motif	5
*Juno*	Mouse/egg	GPI‐anchored, folate receptor family, (protein also known as JUNO)	9
*Cd9*	Mouse/egg	Tetraspanin^b^	11, 12, and 13
*Bouncer*	Zebrafish/egg	GPI‐anchored, Ly6/uPAR superfamily	12
*spe‐9*	Worm/sperm	Single‐pass TM protein with EGF repeats	13 and 14
*spe‐13*	Worm/sperm	Single‐pass TM protein	15; Singson lab unpublished
*spe‐38*	Worm/sperm	Novel four‐pass TM protein	16
*spe‐41/trp‐3*	Worm/sperm	TRP channel	17 and 20
*spe‐42*	Worm/sperm	Six‐pass TM protein with DCSTAMP and Ring‐finger domains	18 and 22
*spe‐49*	Worm/sperm	Six‐pass TM protein with DCSTAMP and Ring‐finger domains	19
*spe‐45*	Worm/sperm	IZUMO1‐like, single‐pass TM protein with Ig‐like domain	20 and 21
*spe‐51*	worm/sperm	Secreted protein with an Ig‐like fold	Mei et al. unpublished
*spe‐36*	Worm/sperm	Secreted protein with an EGF motif	Krauchunas et al. unpublished
*fer‐14*	Worm/sperm	TM protein	26; Kroft et al. unpublished
*egg‐1*	Worm/oocyte	Single‐pass TM protein with LDL receptor repeats	22
*egg‐2*	Worm/oocyte	Single‐pass TM protein with LDL receptor repeats	22

Abbreviations: LDL, low density lipoprotein; TM, transmembrane; TRP, transient receptor potential.

^a^ FIMP1 also exist as a secreted form but only the transmembrane form seems to be responsible for fertility.

^b^ Knockout mice show a severely reduced fertility instead of complete sterility.

Forward genetic screens have been a driving force in identifying the genetic regulation of a biological process.^27^, ^28^, ^29^, ^30^, ^32^, ^33^ Starting with mutants with a phenotype of interest, one can probe the underlying genetic cause and identify complex genetic relationships. Compared to vertebrate models in which forward genetic screens are time‐, cost‐ and labor‐intensive,^34^, ^35^, ^36^ small model organisms have been especially useful for a forward genetic approach in developmental studies.^37^, ^38^ In fact, the majority of the fertilization molecules in *C*. *elegans* were identified through forward genetic screens. In this review, we summarize genetic screening strategies that led to identifying those fertility mutants and provide a perspective for future research that uses genetic approaches in *C*. *elegans*. We also discuss how new technologies such as next‐generation sequencing and genome‐editing tools help us advance our understanding of the genetic regulation of fertility.

## 2 *C*. *ELEGANS* AS A MODEL TO STUDY REPRODUCTION

*C*. *elegans* is a widely used model organism because of its ease of culture, low cost, short life cycle and the availability of genetic tools.^33^ These advantages together with their hermaphroditic mode of reproduction make it relatively convenient to isolate mutants by chemical mutagenesis. Temperature‐sensitive (ts) mutations generally exist more frequently in *C*. *elegans* than in other multicellular organisms.^39^ Additionally, the fertility of the animal is inherently ts, higher at permissive and lower at restrictive temperatures. These features make it possible to find and maintain homozygous fertility mutants. *C*. *elegans* are transparent and so the whole reproductive tract can be observed in live animals. Unfertilized oocytes are readily distinguishable from unhatched embryos, allowing us to tell fertilization defects apart from embryonic lethality.^40^ Hermaphrodites can self‐fertilize to produce self‐progeny but can also be mated with males to produce out‐cross progeny. The hermaphroditic mode of reproduction ensures that sterility in an unmated mutant hermaphrodite is likely due to defects in the gametes and not mating behaviors or copulation. These features make *C*. *elegans* a good model to study fertilization. Studying fertility in *C*. *elegans* as a nematode species will also inform our understanding of reproduction in other nematodes, such as parasitic nematodes that cause human disease or cause losses in both plant and animal agriculture.^41^

**FIGURE 2:**
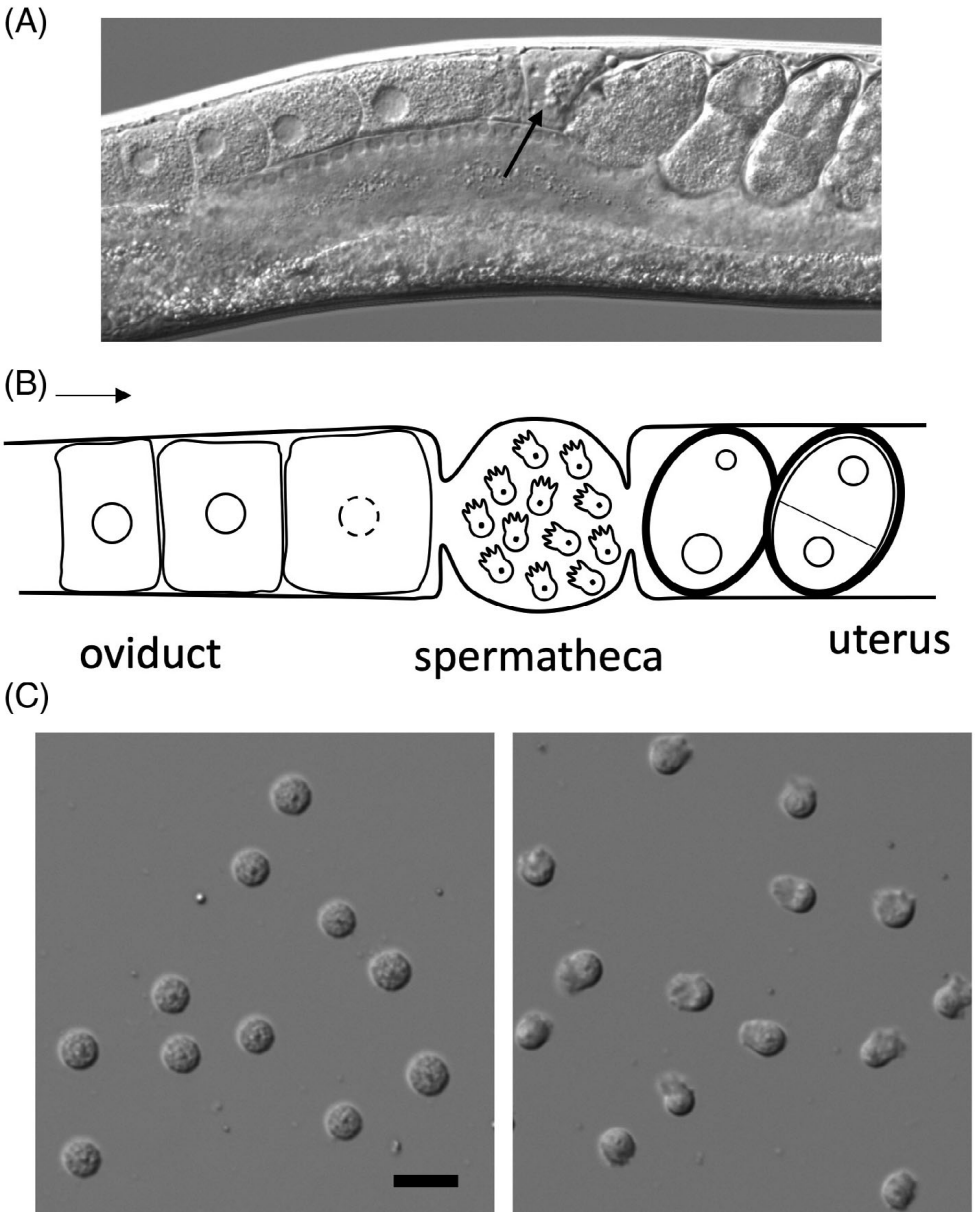
Hermaphrodite reproductive tract and sperm. A, DIC image of a live worm. Themiddle section of the worm is shown here. Arrow is pointing at sperm in the spermatheca. B, A diagram illustrating the middle section of the reproductive tract. Arrow is pointing the direction at which oocytes move. C, Images of spermatids (left) and spermatozoa (right). Scale bar is 10 μm

*C*. *elegans* fertilization takes place in the spermatheca in an assembly‐line fashion.^42^ Oocytes move from distal to proximal gonad as they enter prophase of meiosis I. When oocytes get close to the spermatheca, they receive maturation signals from the major sperm proteins (MSP) secreted by the sperm.^43^ Matured oocytes are ovulated into the spermatheca, fertilized and then pushed into the uterus where they finish meiosis and start embryogenesis (Figure 2A‐B).^42^ Oogenesis starts from young adulthood and continues into later adult stages. Before making oocytes, the germline of a hermaphrodite makes a finite number of spermatids during the last larva stage. The first ovulation pushes the spermatids into the spermatheca, where spermatids undergo a post‐meiotic differentiation process called sperm activation or spermiogenesis.^44^ Activation transforms spherical and non‐motile spermatids into ameboid and motile spermatozoa with pseudopods (Figure 2C ).^40^, ^45^ Motility is important for the sperm: some sperm can be swept out of the spermatheca by passing oocytes and they rely on their motility to crawl back. Defects during spermatogenesis cause sperm to lose the ability to fertilize the egg. The unfertilized oocytes are laid on the plate from the beginning of the reproductive period, in contrast to normal worms that only lay unfertilized oocytes when they are depleted of sperm. Genes that show this oocyte‐laying mutant phenotype of spermatogenesis defects are named “*spe*.” Similarly, genes with mutants showing egg‐sterile (unfertilizable) or egg‐activation defective phenotypes are named “*egg*” (see Box 1 for more naming information).

**Nomenclature of reproductive mutants in *C*. *elegans***

Since the introduction of the *C*. *elegans* model system,^28^ the gene nomenclature for fertility genes that impact sperm or oocyte development or function has changed to more accurately describe mutants and attempt to minimize confusion. The first mutants that impacted sperm development or fertilization function were reported by the Ward Lab^46^, ^47^ and were given the *fer* (FERtilization defective) designation. Additional mutations that impacted sperm (*spe*, SPErmatogenesis defective) as well as mutants that potentially impacted oocyte development (ooc, OOCyte defective mutants) were subsequently reported by the Herman lab.^43^ Because the molecular nature of these mutants was not known at the time, it was felt that the *spe* gene designation was more encompassing than the *fer* designation. It was decided to discontinue using *fer* as a name for subsequent sperm development or fertilization function mutants. Unfortunately, a number of these legacy mutants have been lost over the last 40 years. More recently, the Singson lab introduced the *egg* (EGG sterile) gene designation to describe mutants that impacted egg fertilization or egg activation.^25^, ^49^, ^50^, ^51^ Here we use the term egg to describe a mature (fertilization competent) female gamete or oocyte. The term Ste is widely used to describe a broad spectrum of fertility phenotypes that in most cases do not impact fertilization.

## 3 SCREENING FOR FERTILIZATION MUTANTS: STRATEGIES

**FIGURE 3:**
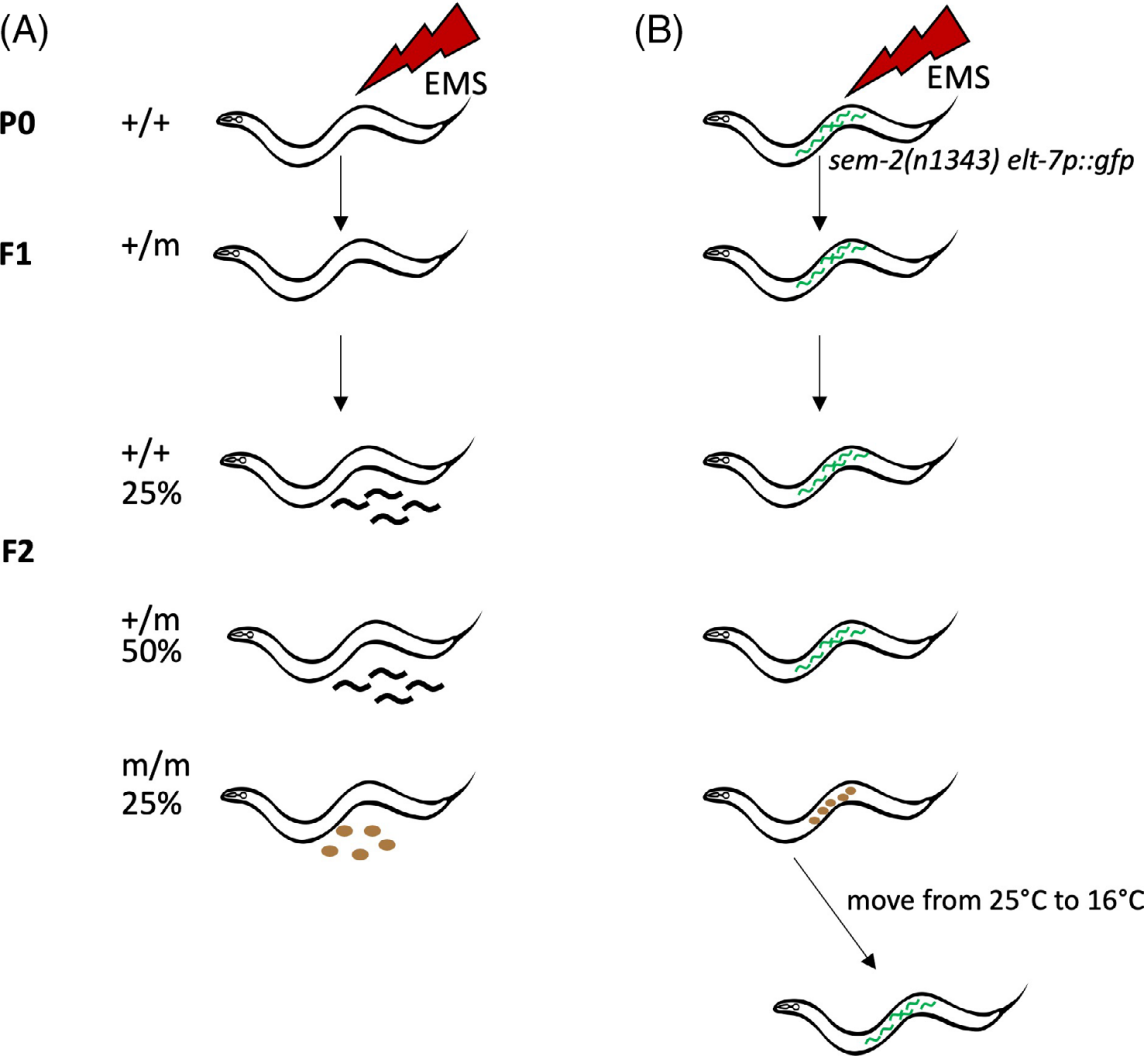
Screening strategies. A, A classic genetic screen looking for recessive mutations that impact fertility. P0 refers to the generation that receives mutagen treatment. F1 and F2 are the first and second generation of progeny. Genotypes of different generations are listed as +/+, +/m, or m/m and their frequencies are also shown. Here “m” represents any mutation in a given locus. Dark squiggly lines represent progeny whereas brown ovals represent unfertilized oocytes. B, Strategy of the screen in the Singson lab. The starter strain carries the*sem‐2*mutation that causes larvae to hatch inside of the mother (green squiggly lines). Candidate sterile F2s (m/m) are grown at 25°C (restrictive temperature) and shifted to 16°C (permissive temperature) to recover fertility. For easy viewing, oocytes and larvae are not drawn to scale

The first report of a forward mutagenesis screen for fertility mutants in *C*. *elegans*, by Hirsh and Vanderslice, came shortly after Sydney Brenner first introduced *C*. *elegans* as an experimental model and described its mutagenesis and genetics.^33^, ^52^ Hirsh and Vanderslice looked for ts sterile mutants in a classic genetic screen (Figure 3A).^38^ Parental worms (P0) were mutagenized by ethyl methanesulfonate (EMS) and allowed to produce F1 and F2 generations of self‐progeny. Individual F2 worms were singled out and allowed to produce the F3 generation at permissive temperature (Table 2). The F3 progeny were then split and some siblings were moved to the restrictive temperature and examined by their phenotype. Any F2 worms that carried a homozygous ts mutation that affects fertility would have all sterile F3s. These lines were identified and propagated from F3 siblings at permissive temperature. Out of ~7700 F2s screened, they identified 223 ts mutants encompassing a broad spectrum of phenotypes, ranging from embryonic‐lethal and developmental mutants, gonadogenesis mutants, to mutants that affect early germline specification. Twenty‐four of these mutants showed a Spe phenotype. Some of these genes were later cloned and other mutants were lost to antiquity. Importantly, their screen demonstrated the possibility of isolating ts mutants for the study of fertility and gametogenesis.

**TABLE 2:** Comparisons of different screening strategies

Screening (reference)	Generation of worms after mutagenesis	Growth temperature of each generation	Treatment of each generation (singled out or not)
Hirsh and Vanderslice 1976 (47)	F1	N/A	No
	F2	16°C	Singled
	F3	25°C	Split (replicated to 25°C)
Argon and Ward 1980 (41)	F1	N/A	Singled
	F2	25°C	Identified by oocyte‐laying phenotype
	F3	16°C	N/A
L'Hernault 1988 second strategy (15)	F1	N/A	Singled
	F2	25°C	No, picked by marker and oocyte‐laying phenotype, mated with wild‐type males
	F3	N/A	N/A
Singaravelu 2015 (20)	F1	N/A	No
	F2	25°C	Only potential sterile mutants are singled out
	F3	16°C	N/A

*Note*: 16°C is permissive whereas 25°C is restrictive temperature.

To further understand sperm motility and fertilization, another screen was performed with small variations from the Hirsh and Vanderslice screen.^46^, ^47^ Instead of transferring all of the F3 generations to restrictive temperature, F1s were singled out and the F2 generation was grown at restrictive temperature (Table 2). Only those F2 populations showing an oocyte‐laying phenotype, a sign that fertilization did not occur, were shifted to permissive temperature. The mutations were recovered by heterozygous F2 siblings if fertility did not recover. Mutants from these two screens include a class of Spe mutants (named Fer, for historical reasons; see Box 1) in which defective sperm contact the oocytes but fail to fertilize them.^46^, ^47^, ^53^ Further analyses suggested that mutant sperm had motility defects that prevented them from crawling back to the spermatheca after being swept by passing oocytes. Most of the Fer mutant sperm made spermatozoa with short or misshaped pseudopods.^53^, ^54^ Together, these Fer mutants demonstrated the power of mutagenesis screens in dissecting the process of spermatogenesis and offered key insights into fertilization and sperm development.

To characterize spermatogenesis, L'Hernault and coworkers designed a screen for *spe* mutants.^17^, ^21^ They described two types of strategies. One strategy is similar to the one described above. All the generations were grown at restrictive temperatures. They allowed several F1s to be picked or laid and grown on a plate. F2s were examined for the appearance of lots of early oocytes. In the other strategy, they used a starter strain with one or multiple morphological markers. Mutagenized P0s were crossed with wild‐type males. The F1 generation were singled out onto individual plates, and F2 larvae were shifted to restrictive temperatures (Table 2). Plates with lots of early unfertilized oocytes were analyzed for linkage and for fertility when outcrossed with wild‐type males. Any mutations that appeared linked with the morphological marker likely affected genes on the same chromosome as the marker. The initial outcross not only helped with establishing linkage but also reduced the number of extraneous mutations thus lowering false‐positive rates. With these strategies, they concentrated on Chromosome I and identified 23 *ts* and non‐conditional mutations belonging to 11 complementation groups. Poisson analysis of mutant frequency suggested that their screen nearly reached saturation for Chromosome I mutations. The phenotypes of these mutants covered various stages of spermatogenesis and sperm functions, including early spermatogenesis, sperm activation, sperm‐oocyte interactions and paternal contribution to embryogenesis.^17^ Among the mutants, the *spe‐9* and *spe‐13* mutants make spermatids that are morphologically normal, can differentiate into spermatozoa, can migrate, but fail to fertilize the oocytes^14^, ^55^ and Krauchunas et al. in preparation). This *spe‐9* class sperm phenotype is seen in both hermaphrodites and males. The gene *spe‐9* encodes a single‐pass transmembrane protein with multiple EGF repeats and is thought to mediate signaling or adhesion with the oocytes.^14^, ^16^ Since *spe‐9* is the first gene found to regulate sperm‐egg interactions, it defines the *spe‐9* class,^56^ which now comprises 10 genes.

To better understand sperm‐oocyte interactions, the Singson lab developed a screening strategy (Figure 3B) based on screens that looked for maternal‐effect embryonic lethal mutants.^57^, ^58^, ^59^, ^60^, ^61^ The goal of this screen is to find ts mutants in which sterility results from defects in sperm‐oocyte interactions. To facilitate mutant selection, we use a starter strain that carries a *sem‐2* mutation and an embryonic gut marker^22^ (Figure 3B). The *sem‐2* mutation leads to defects in muscles that control egg laying but do not affect vulva opening.^59^, ^62^ Fertilized eggs hatch inside of the mother, causing her to form a “bag of worms” and die.^38^ In this genetic background, any sterile F2 worms would appear normal and crawl on the plate whereas fertile worms would form a bag of worms and die. The gut lineage marker *elt‐7p::gfp* is turned on early in the embryo and helps select against any mutants that are maternal‐effect embryonic lethal.^22^ We grow the F2s at restrictive temperature and select candidates that are non‐baggers with a GFP‐negative uterus (Table 2). At this step, we exclude mutants that show obvious defects in gonad development. We then shift candidates to permissive temperature, where only the ts mutants recover fertility. Compared to the Hirsch and Vanderslice screen, this screening strategy eliminates the labor‐intensive step of singling out large numbers of F1 or F2 generations, allowing us to pick sterile worms from a population of fertile ones. However, the use of *sem‐2* mutant background precludes the possibility of only selecting mutants that lay oocytes.

A version of this screen was done with the addition of crossing the sterile F2s with wild‐type males when shifting them to permissive temperature, thus favoring the recovery of *spe* mutations.^22^ This screen identified sperm‐sterile mutants that among others define two key genes that function during sperm‐egg interactions. *spe‐45* encodes a single‐pass transmembrane protein with an Ig‐like domain, similar to mammalian Izumo1.^22^, ^24^ *spe‐51* encodes a secreted molecule with an Ig‐like fold (Mei et al, in preparation). Both mutants show the same *spe‐9* class phenotype: sperm show normal morphology and motility but fail to fertilize the oocytes despite direct contact. The discovery of *spe‐45* and s*pe‐51* added to the collection of 12 sperm‐egg interaction genes identified by us and others (Table 1 and Figure 1B). Of the 10 sperm function genes, two encode secreted and the other eight encode transmembrane proteins. The fact that these genes are required non‐redundantly, suggests that they form a higher‐order complex at the interface between sperm and egg, which we refer to as a fertilization synapse.^2^

It is worth pointing out that the directed fertility screens are not the only ones that recover *spe* or *egg* mutants. Screens that are designed to catch maternal‐effect embryonic lethal mutants or oogenesis mutants have found sterile mutants. Multiple groups have carried out such screens and due to space limits, their screening strategies were not discussed in detail here.^57^, ^58^, ^59^, ^61^, ^63^, ^64^ Sometimes sterile mutants from these screens are shared with us by the community and found to define novel fertility genes (Reference 20 and our unpublished data). Other times sterile mutants were misclassified as embryonic lethal and sometimes discarded. For example, the *spe‐49* gene was initially named *let‐479* because the phenotype was thought to be embryonic lethality.^21^ Therefore, community mutation collections could be a rich source of uncharacterized fertility mutants.

The screen in the Singson lab described above is being continuously performed in our lab, in the hope that we come across fertilization‐defective *egg* mutants. Each time when we perform the screen, instead of attempting to characterize every single mutant, we prioritize our characterization of mutants based on their phenotypes. This allows us to focus on understanding their underlying biology and identifying new genes. Meanwhile, we continue the screen in the lab so that we keep adding new mutants that potentially define new genes. This approach is a move away from the traditional strategy of completing a saturation mutagenesis before moving to any molecular analysis.

## 4 NEW PERSPECTIVES FOR A FORWARD GENETIC APPROACH

We have so far discovered 10 proteins that are required for sperm function during fertilization. What binding partners are on the oocyte surface has long been a question in the field. In *C*. *elegans*, EGG‐1/EGG‐2 is a semi‐redundant pair of LDL receptor repeat‐containing proteins that are required in the oocytes for fertilization.^25^ Originally identified as candidate genes that encode oocyte surface proteins with ligand‐receptor binding domains, EGG‐1/2 were later shown to not bind to SPE‐9 in cultured cells (Singson lab unpublished data). These observations support the hypothesis that additional molecules on the oocyte surface exist to mediate recognition and adhesion with the sperm. One could argue that the reason our screen has recovered far fewer *egg* mutants is the tight link between fertilization and egg activation. It is true that oocyte maturation, fertilization and egg activation are a set of precisely regulated, tightly linked and continuous events, which possibly involve some shared genes and genetic regulation. However, defects in one or more of these events should lead to the same oocyte‐laying phenotype. Thus, the tight link between these events should not interfere with gene discovery by a screen. Because forward genetic screens remain a productive method in discovering missing pieces of this puzzle, we propose modifying our current screening strategy to put more emphasis on egg‐sterile mutants as described above.

Screening for ts mutants of essential genes has been a useful strategy because it allows for easy maintenance of mutants and sometimes offers unique insights of protein functions.^16^ However, ts alleles are relatively rare, with some genes not mutable to a ts phenotype.^65^ Based on our own and others' observations, only 5% to 10% of sterile mutants are ts. Thus, one reason that we see a lot more ts Spe mutants than *egg* mutants could be that the *egg* genes do not tend to mutate to a ts phenotype. Thus, broadening our screening to include non‐conditional mutants might facilitate our search for egg‐sterile mutants. Any potential mutants can be maintained by siblings selection until the mutation is mapped to a chromosomal region so we can use a balancer to make a stable line. Alternatively, we could incorporate a balancer chromosome into the screen and only search for egg‐sterile mutants in the region covered by the balancer.

Searching for the egg‐sterile mutants may be confounded by the fact that sterility can be cause by a broad spectrum of defects such as in the gonad and in germline development and gametogenesis. Among these sterile mutants are all of the *spe* mutants that show the same oocyte‐laying phenotype as any potential egg‐sterile mutants. These *spe* mutations are carried at a high frequency in the F1 population of mutagenized parents, up to one in 30 independent F1s.^17^, ^66^ Thus, it is imperative to further improve our strategy of mutant selection. To avoid selecting *spe* mutants, we can test for fertility rescue by mating the mutants with wild‐type males. A recovery of fertility would suggest the mutant is a *spe*. Moreover, molecular markers that label the sperm and oocytes can be used to help select sterile worms that have good‐looking sperm and oocytes. For example, a germline specific cell membrane marker and/or a histone marker will allow us to observe the morphology of gametes. These markers together with microscopy will also help filter out gametogenesis and embryonic lethal mutants.

## 5 LIMITATIONS OF FORWARD GENETIC SCREENS

Although a powerful and unbiased way to identify genetic regulation of biological processes, a forward genetic method has its own limitations. A blind spot of forward genetic screening is functional redundancy, where paralogous genes have overlapping functions. Loss of one paralog often is not sufficient to cause a phenotype, due to compensation by another paralog. In this case, these genes could be identified only through certain dominant alleles.^63^, ^67^, ^68^, ^69^, ^70^ Multiple examples of redundant genes exist in *C*. *elegans* spermatogenesis,^71^, ^72^ oogenesis,^67^, ^68^ ovulation,^43^ fertilization,^25^ and oocyte‐to‐embryo transition^49^, ^50^ and these genes were identified through reverse genetic or biochemical approaches. It is estimated that 30% of *C*. *elegans* genome encode proteins with one or more paralogs.^73^, ^74^, ^75^ Although it is not known to what extent these paralogs have redundant functions, this level of redundancy poses a challenge in gene discovery with forward genetic approaches.

Other than redundancy, pleiotropy is another potential limitation of forward genetic screens. One gene product may regulate multiple processes, with different timing or in different tissues. Phenotypes of loss of function alleles may represent only one specific function but mask others. An example of this redundancy during *C*. *elegans* spermatogenesis is *spe‐6*. Loss‐of‐function alleles showed that SPE‐6 played roles in completing meiosis and organizing and assembling the sperm cytoskeleton MSPs during early spermatogenesis.^76^ However, hypomorphic alleles of *spe‐6* revealed its later role in coordinating sperm activation.^77^ Similar to redundancy, pleiotropic genes could be uncovered during a forward genetic screen only by rare and specific alleles.

## 6 TECHNOLOGY ADVANCEMENTS THAT FACILITATE GENE DISCOVERY

Technological advances in recent years, such as high‐throughput sequencing and genome editing tools, have greatly facilitated our molecular identification of genes and characterization of gene functions. Here we describe how these technologies benefit our research and open up opportunities to use other methods to complement forward genetic approaches.

Whole‐genome sequencing (WGS) has totally transformed the way to identify a causative mutation in a given mutant. Before WGS was widely used, genes were cloned by tedious two‐ and three‐ point mapping and SNP (Single Nucleotide Polymorphism) mapping. A mapping‐by‐sequencing approach that couples WGS with genome‐wide SNP mapping^78^, ^79^ has greatly facilitated gene cloning.^80^, ^81^ In this method, a mutant is crossed with a polymorphic strain to produce the F1 and F2 generation. F2s are selected for the mutant phenotype and subjected to WGS. The region where the mutation lies should be enriched for polymorphic markers from the background strain. This method is sensitive, and quickly narrows down the mutation to a few locations thus greatly shortening the time it takes to pinpoint the affected gene.

Another advancement that brings changes to our query into fertilization molecules is in transcriptomics. In *C*. *elegans*, germline‐enriched and/or sex‐biased gene expression profiles have been reported.^82^, ^83^, ^84^, ^85^, ^86^, ^87^ Using mutants in the sex‐determination pathway that cause the hermaphrodites to only produce sperm or oocytes, these transcriptomic datasets yield lists of genes that are specifically expressed in the sperm vs oocytes.^82^, ^83^, ^84^ The availability of transcriptomics data has fueled reverse genetic approaches that overcome some of the limits of genetic screens. Several genes during *C*. *elegans* fertilization and egg activation were identified through reverse genetic approaches, such as *spe‐45* and *egg‐1/egg‐2* that regulate fertilization, and *egg‐3* and *egg‐4/egg‐5* that regulate egg activation.^24^, ^25^, ^49^, ^50^, ^51^ The improved sample processing methodology and increased sequencing sensitivity means that we can look at global transcription in a finer temporal and spatial scale, even at a single‐cell level.^85^, ^86^, ^87^ Due to constraints on sequencing sensitivity, some subtle or transient changes in gene expression may be lost.^87^ Yet, as the technology evolves, this method may strengthen our ability to discover genes that regulate fertilization and egg activation.

The Crispr‐based genome editing tools have expanded our abilities to access protein functions by genetics.^88^, ^89^ Crispr‐based methods have made it more efficient to generate null and conditional alleles, to engineer customized point mutations, and to tag endogenous proteins.^90^, ^91^, ^92^, ^93^ The mouse fertilization molecule SOF1 was identified in an effort to test the functions of a number of testis‐specific genes using Crispr‐based knockout alleles.^5^, ^94^, ^95^ In *C*. *elegans*, some groups have reported using Crispr to examine the functions of genes that regulate fertility.^96^, ^97^ A few groups have had success in tagging and visualizing proteins that exert functions in the germline.^98^, ^99^ Sperm proteins especially those that function at fertilization are usually expressed at low levels that makes visualization challenging. Crispr‐based editing provides a yet another way of localizing proteins in addition to traditional antibody‐based fluorescent labeling. Overall this genome editing tool when combined with transcriptomic datasets will allow us to evaluate gene functions, genetic relationships and protein dynamics at an unprecedented level.

Although genetics‐based fertility gene discovery has been fruitful, biochemical and proteomic studies are also viable approaches. For *C*. *elegans*, both sperm and oocytes can be isolated in bulk as starting material for biochemical analysis.^100^, ^101^ These purifications methods can be used with mutants of *C*. *elegans* that only make sperm or oocytes, which will be powerful in determining the sperm or oocyte proteome. Several proteomics studies were published before, some of which generate gene/protein lists that are relevant for our search of fertilization molecules.^72^, ^102^, ^103^ In addition to purifying whole sperm or oocytes, isolating subcellular compartments such as the MOs of the sperm for proteomics will provide insights into specific proteins that are transported to the surface of the sperm during activation. Additionally, cultured Drosophila S2 cells can be used as a system to express worm proteins for other biochemical assays. Many *C*. *elegans* biologists have a preference for genetic analysis. However, it should be remembered that many well‐worn and newly developed biochemical and proteomic methodologies will be important for both additional gene discovery and gaining molecular mechanistic insights into fertilization.

The SPE molecules at the fertilization synapse discovered thus far, likely represent only part of the picture. Mechanistically, how they interact with one another and with potential partners on the oocyte surface is still elusive. Protein interaction studies can help us identify these interactions. As a community resource, our lab has established the SPE interactome using a membrane yeast two hybrid system.^104^ This interactome offers some insights into the landscape of the sperm surface. For example, SPE‐38 is a four‐pass transmembrane protein of the *spe‐9* class and is required for the correct localization of another component, SPE‐41, at the sperm surface.^18^, ^19^ We hypothesize that it may play a similar role as the tetraspanin CD9 on the mammalian egg surface, serving as a molecular raft organizing other molecules.^11^, ^12^, ^115^ Consistent with this hypothesis, SPE‐38 has been shown to interact with multiple SPE proteins in our interactome.^104^ As more mutants are discovered and the molecular identities of the genes become known, the interactome will continue to grow and give us the opportunity to generate new hypotheses.

## 7 FERTILIZATION SYNAPSE IN NEMATODE PROVIDES INSIGHTS INTO THAT OF MAMMALS

The *spe‐45* gene in *C*. *elegans* was identified not only in our mutant hunt, but also independently by a reverse genetic approach searching for single‐Ig domain containing, sperm‐surface molecules like IZUMO1.^24^ In addition to identical phenotypes of in both the worm and mouse, chimeric SPE‐45 with the mouse IZUMO1 Ig domain expressed as a transgene partially rescued the *spe‐45* mutant fertility, suggesting conserved functions of the Ig domain.^24^, ^105^ The discovery of *C*. *elegans spe‐51* as an Ig‐like fold containing protein further highlights important functions of Ig domains at the fertilization synapse. The fact that shared protein domains are utilized to achieve the same reproductive goal in two evolutionarily distant species suggests a deep conservation of protein structures at the gamete interface despite rapid co‐evolution of gamete‐interacting proteins.^106^, ^107^

The complexity of the fertilization synapse on the sperm side in *C*. *elegans* implies that a great deal of information is still unknown for all species, which may be even more complex.^2^ Recent identification of four sperm surface proteins, SOF1, SPACA6, TMEM95 and FIMP1 in mice supports the complexity of the mouse fertilization synapse.^5^, ^6^, ^7^, ^9^ Loss of functions for each of these molecules does not affect the level or localization of IZUMO1, suggesting a hierarchy in the assembly and dynamics of the fertilization synapse. Similarly, in *C*. *elegans*, loss of certain components of the fertilization synapse does not necessarily affect the localization of others suggesting differential roles of these molecules during gamete interactions.^19^, ^108^ These observations lead us to think that proteins on the sperm side of the synapse may or may not each have their own binding partner on the egg surface, with some of them playing a scaffolding rather than a direct interacting role. In mice, the JUNO‐IZUMO1 interaction is not sufficient for sperm‐egg fusion, as shown by cell binding assays and structural analyses, suggesting additional molecules are involved.^109^, ^110^, ^111^, ^112^, ^113^ In *C*. *elegans*, additional egg surface receptors are also to be identified and these questions will be the focus of future research.

## 8 CONCLUSIONS

Our ongoing journey using *C*. *elegans* as a paradigm to understand the fertilization process has complemented our understanding of mammalian fertilization. Our findings also provide future perspective on the control of reproduction in other species including parasitic nematodes. We argue that robust efforts in a variety of model systems will be the most effective way to understand nature's mysteries of conception. Forward and reverse genetic approaches to study reproductive processes have been reported for fruit fly,^114^ zebrafish^115^ and mice.^116^, ^117^, ^118^ Although these efforts in these diverse model systems have identified interesting mutants that impact stem cell biology, meiosis and gametogenesis, we hope that they continue to identify key molecules of fertilization. With the advancement of new technologies, our forward screens, complemented by a reverse genetic and biochemical approaches, will give us a better understanding of the protein interactions at the fertilization synapse, and a better picture about fertilization and fertility in *C*. *elegans* and other animals including humans.

## AUTHOR CONTRIBUTIONS

**Xue Mei:** Conceptualization; formal analysis; writing‐original draft; writing‐review and editing. **Andrew W. Singson:** Conceptualization; funding acquisition; investigation; project administration; supervision; writing‐review and editing.

## CONFLICT OF INTEREST

The authors declare no potential conflict of interest.

### PEER REVIEW

The peer review history for this article is available at https://publons.com/publon/10.1002/ggn2.10034.

[Correction added on 16 January 2021, after first online publication: Peer review history statement has been added.]

## Data Availability

Data sharing is not applicable to this article as no new data were created or analyzed in this study.
